# Induction of RIPK3/MLKL-mediated necroptosis by Erigeron breviscapus injection exhibits potent antitumor effect

**DOI:** 10.3389/fphar.2023.1219362

**Published:** 2023-06-16

**Authors:** Xiuping Guo, Rui Li, Jinjin Cui, Chujuan Hu, Haoyang Yu, Ling Ren, Yangyang Cheng, Jiandong Jiang, Xiao Ding, Lulu Wang

**Affiliations:** ^1^ Institute of Medicinal Biotechnology, Chinese Academy of Medical Science and Peking Union Medical College, Beijing, China; ^2^ State Key Laboratory of Phytochemistry and Plant Resource in West China, Kunming Institute of Botany, Chinese Academy of Sciences, Kunming, China

**Keywords:** Erigeron breviscapus injection, Dengzhanxixin, *Erigeron breviscapus (Vant.) Hand.-Mazz*, colorectal cancer, necroptosis, drug resistance

## Abstract

Colorectal cancer (CRC) is the second leading cause of tumor-related deaths worldwide. Resistance of tumor cells to drug-induced apoptosis highlights the need for safe and effective antitumor alternatives. Erigeron breviscapus (Dengzhanxixin in China) injection (EBI), extracted from the natural herb *Erigeron breviscapus (Vant.) Hand.-Mazz* (EHM), has been widely used in clinical practice for cardiovascular diseases. Recent studies have suggested that EBI’s main active ingredients exhibit potential antitumor effects. This study aims to explore the anti-CRC effect of EBI and elucidate the underlying mechanism. The anti-CRC effect of EBI was evaluated *in vitro* using CCK-8, flow cytometry, and transwell analysis, and *in vivo* through a xenograft mice model. RNA sequencing was utilized to compare the differentially expressed genes, and the proposed mechanism was verified through *in vitro* and *in vivo* experiments. Our study demonstrates that EBI significantly inhibits the proliferation of three human CRC cell lines and effectively suppresses the migration and invasion of SW620 cells. Moreover, in the SW620 xenograft mice model, EBI markedly retards tumor growth and lung metastasis. RNA-seq analysis revealed that EBI might exert antitumor effects by inducing necroptosis of tumor cells. Additionally, EBI activates the RIPK3/MLKL signaling pathway, a classical pathway of necroptosis and greatly promotes the generation of intracellular ROS. Furthermore, the antitumor effect of EBI on SW620 is significantly alleviated after the pretreatment of GW806742X, the MLKL inhibitor. Our findings suggest that EBI is a safe and effective inducer of necroptosis for CRC treatment. Notably, necroptosis is a non-apoptotic programmed cell death pathway that can effectively circumvent resistance to apoptosis, which provides a novel approach for overcoming tumor drug resistance.

## 1 Introduction

Cancer is a significant global threat to human life worldwide. The Global Cancer Statistics Analysis Report of 2020 revealed that there were 1.9 million new cases and 0.9 million mortalities associated with colorectal cancer (CRC), making it the second leading cause of cancer-related deaths (Sung et al., 2021). Although marked progress has been made in the early diagnosis and treatment of CRC, the 5-year survival rate for metastatic CRC remains low, at only 14%. While surgical intervention is the optimal treatment for CRC, the difficulty of surgical intervention increases with the progression of CRC, especially in the late stages (Dekker et al., 2019), posing significant challenges to clinical management (Biller and Schrag, 2021). Additionally, chemotherapy can cause severe adverse reactions due to its systemic toxicity, and targeted and immune therapies face challenges in addressing the heterogeneity of CRC. Moreover, drug resistance gradually develops during the treatment process, thereby promoting tumor recurrence and metastasis (Keum and Giovannucci, 2019). Therefore, there is an urgent need for safe and effective anti-CRC drugs.

Erigeron breviscapus, commonly known as Dengzhanxixin in China, is a dried medicinal herb obtained from the plant species *Erigeron breviscapus (Vant.) Hand.-Mazz* (EHM). The earliest documented utilization of EHM can be found in the “Compendium of Materia Medica from Yunnan South,” a pharmacological book compiled by Lan Mao during the rule of the Ming dynasty in China (AD1368-1644). Erigeron breviscapus injection (EBI) contains 9 flavonoid and 31 phenolic acid compounds (Wang et al., 2010), among which scutellarin (SCU) and dicaffeoylquinic acid esters (including 3,5-dicaffeoylquinic acid, 3,4-dicaffeoylquinic acid, 4,5-dicaffeoylquinic acid, 3,4,5-tricaffeoylquinic acid) are the main active ingredients. EBI exhibits pharmacological effects of promoting blood circulation, removing blood stasis, dredging collaterals, and relieving pain (Sun and Zhao, 2009). It has been widely used in the treatment of ischemic stroke, coronary heart disease, and angina, and is recorded in the 2020 edition of Pharmacopoeia of the People’s Republic of China (ChP 2020) (Wang et al., 2018; Dong and Qu, 2022). Recent studies have suggested that the active ingredients in EBI have potential antitumor effects. SCU has been shown to have potent antitumor properties against various types of cancer, such as non-small cell lung cancer (NSCLC) (He et al., 2021), colorectal cancer (Tan et al., 2020), kidney cancer (Deng et al., 2018), bladder cancer (Lv et al., 2019), and liver cancer (Ke et al., 2017), due to its ability to arrest the cell cycle, induce cell apoptosis, inhibit cell proliferation, and reverse epithelial-mesenchymal transition. Moreover, caffeoylquinic acid esters have been shown to promote cancer cell apoptosis, suppress tumor growth, and hinder metastasis through diverse molecular mechanisms (Puangpraphant et al., 2011; Zhou et al., 2020). These findings suggest that EBI might hold promise in inhibiting CRC progression.

Inducing cell apoptosis is a vital therapeutic strategy for managing the excessive proliferation of cancer cells. However, various mechanisms can lead to cancer cells developing resistance to apoptosis (Sharma et al., 2019). Recent research has highlighted the dysregulation in the balance between pro- and anti-apoptotic proteins in CRC (Carr et al., 2016), along with the downregulation and functional loss of death receptors, leading to impaired extrinsic apoptotic signaling pathways (Zins et al., 2007; Hoogwater et al., 2010). Necroptosis is a non-apoptotic programmed cell death type that is mediated by death receptors, principally involving receptor-interacting protein kinase 1 (RIPK1), receptor-interacting protein kinase 3 (RIPK3), and mixed lineage kinase domain-like protein (MLKL) in the signal transduction cascade (Weinlich et al., 2017). Mounting evidence suggests that necroptosis is meticulously regulated, with these proteins coordinating the formation of different protein complexes, including the phosphorylation of RIPK3, which in turn triggers the phosphorylation and oligomerization of MLKL. Subsequently, MLKL translocates to the membrane and disrupts its integrity, ultimately leading to cell death (Weinlich et al., 2017). Studies have shown that the expression of RIPK1 and RIPK3 is significantly lower in CRC tissue than in surrounding normal tissues (Moriwaki et al., 2015). Moreover, the decreased expression of RIPK3 and MLKL in tumors is markedly associated with poorer overall survival rates (Li et al., 2017; Yan et al., 2018). Considering that necroptosis mechanisms and signal regulation differ from apoptosis, inducing necroptosis represents a novel approach to identify molecular targets and develop therapeutic strategies for the treatment of CRC.

Natural products provide a unique source of compounds for the development of anticancer drugs. Several natural compounds such as camptothecin, paclitaxel, and vinblastine have already been established as successful anticancer drugs (Lin et al., 2020). In addition to their direct killing effect, an expanding body of research indicates that natural products have the potential to trigger multiple forms of programmed cell death, making them promising alternatives for cancer treatment. For example, curcumin and its analogs such as dimethoxycurcumin and curcuminoid B63 have been demonstrated to induce paraptosis in gastric cancer cells (Millimouno et al., 2014). Artesunate, a water-soluble derivative of artemisinin, has been reported to induce lysosomal iron-dependent cell death in pancreatic ductal adenocarcinoma cells (Eling et al., 2015), while metformin, a derivative of galegine, can induce pyroptosis in esophageal squamous cell carcinoma cells (Wang et al., 2019). Notably, scutellarin, the primary constituent of erigeron breviscapus, has been shown to stimulate apoptosis in cancer cells and boost cisplatin-induced autophagy through the c-met/Akt signal pathway, counteracting NSCLC’s resistance to cisplatin (Sun et al., 2018). However, to date, the mechanism of EBI to induce cancer cell death has not been systemically studied.

Considering that EBI is a clinically approved drug with minimal toxicity and potential antitumor properties, we conducted experiments to investigate its effectiveness against CRC and the underlying mechanisms. Our study confirmed for the first time that EBI can inhibit the growth and metastasis of CRC both *in vitro* and *in vivo*, primarily by inducing necroptosis in cancer cells. These findings provide novel insights into the potential of necroptosis induction as a treatment strategy for cancer and provide a basis for further development of EBI as an effective and low-toxicity anticancer drug.

## 2 Materials and methods

### 2.1 Chemicals

Erigeron breviscapus injection (EBI) was obtained from the Yunnan Biovalley Pharmaceutical Co., Ltd (Yunnan, China), according to the ChP 2020, the content of the main active ingredients in EBI is 2.0 mg/mL. GW806742X (10 mM in DMSO) was purchased from MedChem Express (United States).

### 2.2 Cell lines and cell culture

Human SW620, Caco2, HT29 cells were purchased from the American Type Culture Collection (ATCC, MD, United States) and cultured in RPMI 1640, MEM and McCoy’s 5A medium (Gibco, United States), respectively. The growth medium was supplemented with 10% fetal calf serum (FBS, Gibco, United States), streptomycin (100 ug/mL) and penicillin (100 U/mL) (Gibco, United States), and the cells were incubated at 37°C in a humidified incubator containing 5% CO_2_.

### 2.3 Cell viability assay

Cell viability was assessed using a CCK-8 assay kit (Beyotime Biotech. Inc., Shanghai, China) according to the manufacturer’s instructions. Briefly, SW620, Caco2, HT29 cells were seeded in 96-well plates at a density of 1 × 10^4^ cells/100 μL of complete culture medium and cultured at 37°C in a humidified incubator containing 5% CO_2_. After 24 h of incubation, all cells were treated with blank or varying concentrations (6.25, 12.5, 25, 100, 160, 200, 300, and 400 μg/mL) of EBI for 24, 48, or 72 h. At different time points, the medium was removed, and CCK-8 reagent was added to each well, and the plates were further incubated for 2 h. Absorbance at 450 nm was measured using a plate-reader (BioTek, Vermont, United States). Specially, SW620 cells were pretreated with 1 μM GW806242X (MedChemExpress, United States) 1.5 h prior to EBI addition to evaluate the cell viability and IC50 in the presence of necroptosis inhibitor.

### 2.4 Flow cytometry analysis

Cell death were assessed by flow cytometry analysis of annexin V and propidium iodide (PI) using the FITC Annexin V Apoptosis Detection Kit I from BD Biosciences (United States) according to the manufacturer’s instructions. Briefly, SW620 cells were seeded in 6-well plates at a density of 1 × 10^6^ cells/well and cultured at 37°C in a humidified incubator containing 5% CO_2_ for 24 h. After 24 h of incubation, SW620 cells were treated with either blank or varying concentrations (10, 15, 20, 40, and 100 ug/mL) of EBI. After 12 h, the cells were trypsinized, transferred to a tube containing the complete culture medium. The cells were then centrifuged at 1,000 rpm for 5 min, washed with cold PBS and resuspended in ×1 Binding Buffer. FITC Annexin V and PI were added to each tube and incubated for 15 min at room temperature in the dark. The percentage of apoptotic or necrotic cells was analyzed using a CytoFLEX S (Beckman Coulter, Inc., United States). Specially, cells treated with 40 μg/mL of EBI were pretreated with 1 μM GW806242 × 1.5 h prior to EBI addition, while an equal volume of dimethyl sulfoxide was applied as the control.

### 2.5 Migration and invasion assay

Cells were subjected to either blank or 10, 15, 20, and 40 μg/mL EBI for 24 h, trypsinized and resuspended in medium supplemented with 1% FBS. For the migration assay, 3 × 10^5^ cells were seeded into the upper chamber of transwell inserts (Corning Costar, MA, United States) with a porous polycarbonate membrane (8 μm pore diameter), while the lower chamber contained 800 μL of medium supplemented with 20% FBS. After incubation for 30 h, non-migrating cells on the upper surface of the membrane filters were removed by mechanical means using cotton swabs. The migrated cells on the lower side of the filter were fixed with cold 4% paraformaldehyde (PFA), stained with 0.5% crystal violet solution for 2 h, washed with PBS, and imaged using a microscope. For the invasion assay, the upper chamber of the transwell was coated with 70 µL of Matrigel (1:20, Corning Costar, MA, United States). The cells that invaded the lower surface of the membrane filter were fixed with cold 4% PFA, stained with 0.5% crystal violet solution, washed with PBS, and then imaged as described above.

### 2.6 Cell morphological analysis

To assess the effects of EBI on the morphological characteristics of SW620 cells, microscopy (at the magnification of ×100 and ×200) was utilized. SW620 cells were seeded in 12-well plates at a density of 5 × 10^5^ cells/well and cultured at 37°C in a humidified incubator containing 5% CO_2_ for 24 h. After 24 h of incubation, SW620 cells were treated with blank or vary concentrations (10, 15, 20, 40, and 100 μg/mL) of EBI for 12 h. Three fields in each group were observed, and representative images were shown. Notably, cells treated with 20 μg/mL of EBI were pretreated with 1 μM GW806242 × 1.5 h prior to EBI addition, while an equal volume of dimethyl sulfoxide was applied as the control.

### 2.7 Intracellular ROS measurement

The total intracellular ROS levels were determined by staining cells with dichlorodihydrofluorescein diacetate (DCFH-DA, Beyotime) according to the manufacturer’s instructions. Briefly, SW620 cells were seeded in 12-well plates at a density of 5 × 10^5^ cells/well and cultured at 37°C in a humidified incubator containing 5% CO_2_ for 24 h. After 24 h of incubation, SW620 cells were treated with blank or 20 ug/mL of EBI. At different time points (1, 2, and 4 h), the cells were trypsinized and transferred to a tube along with the complete culture medium. Cells were centrifuged at 1,000 rpm for 5 min, washed with PBS. DCFH-DA was diluted in FBS-free RPMI 1640 medium at 1:1000 to 10 μM, then cells were resuspended with 10 μM DCFH-DA and incubated for 20 min at 37°C in a humidified incubator. The cells were then washed three times with PBS and analyzed using a CytoFLEX S (Beckman Coulter, Inc., United States). To capture the images, the treated cells was incubated with 10 μM DCFH-DA in 12-well plates for 20 min at 37°C in a humidified incubator, and then gently washed with PBS. A fluorescence microscope (Olympus, Japan) was used for optical imaging.

### 2.8 Western blot analysis

The cells were lysed with RIPA buffer (CoWin Biotech, Jiangsu, China) containing phosphatase inhibitor cocktail and protease inhibitor (CoWin Biotech, Jiangsu, China). The protein lysates were cleared by centrifugation at 12,000 rpm for 30 min at 4°C, and the supernatant fractions were used for Western blot analysis. Protein concentrations were determined using the bicinchoninic acid (BCA) protein assay kit (Thermo Fisher Scientific, United States). The protein samples were denatured at 95°C for 10 min with ×5 loading buffer (CoWin Biotech, Jiangsu, China) and separated by 10% SDS-PAGE gels before being blotted onto PVDF membranes. The membranes were blocked with 5% Bovine Serum Albumin (BSA, Sigma, United States) in tris-buffered saline-Tween 20 (TBST) for 1 h at room temperature, and incubated with primary antibodies at 4°C overnight. The primary antibodies used were anti-MLKL (ab184718), anti-RIPK3 (phospho S227, ab209384) purchased from Abcam (United Kingdom), anti-MLKL (phospho S358, PA5-105678) purchased from Invitrogen (Thermo Fisher Scientific, United States), anti-RIPK3 (17563-1-AP) and anti-GAPDH antibody (60004-1-Ig) purchased from Proteintech (United States). Subsequently, the membranes were probed with horseradish peroxidase (HRP)-conjugated secondary antibodies (Beyotime, Jiangsu, China) for 1 h at room temperature. Finally, the proteins of interest were analyzed using the ECL Western blotting substrate (Tannon, Shanghai, China).

### 2.9 Small interfering RNA (siRNA) transfection

Transfection of siRNA was carried out using the riboFECT CP Transfection Kit (C10511-05) from Guangzhou RiboBio Co., Ltd. (China), following the manufacturer’s instructions. Briefly, SW620 cells were seeded in 12-well plates at different densities and cultured for 24 h. Con-siRNA (siN0000001-1-5, RiboBio, Guangzhou, China) or MLKL-siRNA (siG08121113901-1-5, RiboBio, Guangzhou, China) at varying concentrations were used for transfection. The following conditions were employed: Condition 1, 1 × 10^6^ cells with 50 nM siRNA; Condition 2, 5 × 10^5^ cells with 50 nM siRNA; Condition 3, 1 × 10^5^ cells with 50 nM siRNA; Condition 4, 1 × 10^6^ cells with 30 nM siRNA; Condition 5, 5 × 10^5^ cells with 30 nM siRNA. Condition 1, which exhibited the highest transfection efficiency, was selected for the subsequent experiments.

### 2.10 Reverse transcription-quantitative PCR (RT-qPCR) analysis

Total RNA was isolated from cells using a PureLink™ RNA Mini Kit (Thermo Fisher Scientific, United States) according to the manufacturer’s instructions. The purified RNA (1 μg) was reverse transcribed into cDNA using the HiFiScript cDNA Synthesis Kit (CoWin Biotech, Jiangsu, China). Real-time quantitative PCR (qPCR) was performed to evaluate the expression of MLKL using KAPA SYBR FAST qPCR Master Mix (2X) (KK4601, Kapa Biosystems, United States) on a 7,500 Fast Real-Time PCR system (Thermo Fisher Scientific, United States). The experiment was conducted in triplicate, and the expression levels were normalized to GAPDH. The following primer sequences were used for qPCR: MLKL-F, AGG​AGG​CTA​ATG​GGG​AGA​TAG​A; MLKL-R, TGG​CTT​GCT​GTT​AGA​AAC​CTG; GAPDH-F, TCA​AGA​AGG​TGG​TGA​AGC​AG; and GAPDH-R, AGC​CAA​ATT​CGT​TGT​CAT​ACC.

### 2.11 Tumor-bearing mice and treatment

Male nude mice (BALB/c-nu), aged 5–6 weeks and weighing 25 g, were purchased from HFK Bio-Technology.co., LTD (Beijing, China) and were maintained in pathogen-free conditions in accordance with state regulations for animal care. All experimental animal procedures were approved by the Animal Experimental Ethics Committee of the Institute of Medicinal Biotechnology, Chinese Academy of Medical Sciences and Peking Union Medical College. To establish the xenograft human tumor model in mice, 2 × 10^6^ SW620 tumor cells (in 100 μL Matrigel-containing culture medium) were subcutaneously inoculated into the right flank of mice. The tumor-bearing mice were randomly allocated to vehicle control or treatment groups (*n* = 6) before treatment initiation, and treatment began at a mean tumor burden of 150 mm^3^. Vehicle control (saline), EBI (6.4, 12.8, and 25.6 mg kg^−1^) were administered intraperitoneally (i.p.) every day for 15 days. The body weight and tumor volume were monitored every 2 days, and the tumor volume (V) was calculated using an electronic caliper according to the formula:

$$V=\left(L\times W\times W\right)/2$$

Where L (length) is the largest diameter and W (width) is the smallest diameter. Tumor-bearing mice were euthanized at day 24 (1 day after the last administration), and serum samples were collected before sacrifice. Tumor tissue samples and major organs (liver, spleen, lung, and kidney) were also collected for further research. The levels of serum aspartate aminotransferase (AST), alanine aminotransferase (ALT), urea nitrogen (Urea), and creatinine (Crea) were evaluated using a fully automated biochemical analyzer (HITACHI, Japan).

### 2.12 Histology analysis

Tumor tissue samples and major organs (liver, spleen, lung, and kidney) were fixed in 4% PFA for 24 h, embedded in paraffin blocks, and sectioned into 5 μm-thick serial sections. Before staining, the tumor sections were deparaffinized and rehydrated. They were then incubated with a blocking solution for 1 h at room temperature, followed by staining with primary antibodies against p-MLKL or Ki67. Signal development was achieved using Vectastain ABC Elite kit (Vector Laboratories, United Kingdom), and the slides were counterstained with hematoxylin (Vector Laboratories, United Kingdom). The major organs and tumor sections were stained with hematoxylin and eosin (H&E) as previously described (Fischer et al., 2008). After staining, an optical microscope (Olympus, Japan) was employed for optical imaging.

### 2.13 RNA-seq gene expression profiling

The SW620 tumors were collected 1 day after administration of either saline or 25.6 mg kg^−1^ EBI, frozen in liquid nitrogen, and stored at −80°C until further analysis. The total RNA was extracted using TRIzol (Thermo Fisher Scientific, United States) according to the manufacturer’s instructions. The extracted RNA was used to prepare libraries using the NEBNext Ultra RNA Library Prep Kit for Illumina (NEB), following the manufacturer’s protocol. Index codes were attached to identify samples, and Cutadapt (v.2.6) was employed to remove adapters. Clean reads were then mapped to the human reference genome (GRCh38) using Hisat2 (2.1.0) with default parameters. Gene expression was quantified using featureCounts (1.6.4), and mRNA expression z-scores were calculated based on fragments per kilobase of transcript per million mapped reads (FPKM). Heatmaps were generated using pheatmap (v.1.0.12) in R (3.6.1). Differential expression analysis was performed using DESeq2 (v1.4.5) with a *p*-value threshold of ≤0.05. The annotated differentially expressed genes were subjected to Gene Ontology (GO) and Kyoto Encyclopedia of Genes and Genomes (KEGG) enrichment analysis using the Phyper tool, and the Sankey bubble plot was generated using the ggalluvial package.

### 2.14 Statistical analysis

All data were analyzed and graphed using GraphPad Prism software (version 9.5.0). Statistical comparisons were conducted utilizing two-tailed Student’s *t* test, one-way analysis of variance (ANOVA) when appropriate. The present quantified data shown were obtained from at least three independent experiments and expressed as mean ± SEM. *p*-value <0.05 was considered statistically significant.

## 3 Results

### 3.1 Effects of EBI on the growth and cell death on CRC cell lines

**FIGURE 1:**
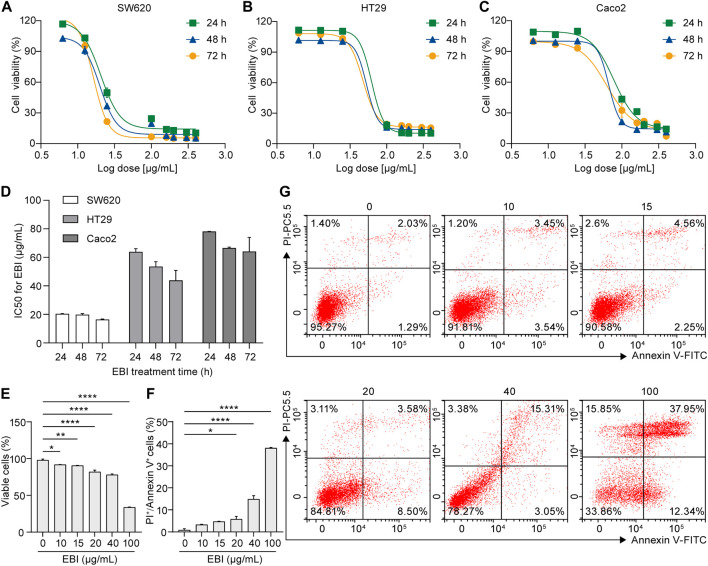
EBI inhibits proliferation and facilitates cell death in human CRC cell lines. **(A–C)** Effect of EBI on the viability of human CRC cell lines **(A)** SW620, **(B)** HT29, and **(C)** Caco2. **(D)** The half-maximum inhibitory concentration (IC50) values (μg/mL) of EBI against different CRC cell lines for 24, 48, or 72 h treatments. Mean ± SEM. **(E–G)** Flow cytometric analysis of annexin V and PI staining of SW620 cells. The quantitative analysis of viable cells **(E)** and quantitative analysis of double-positive cells for annexin V and PI **(F)**. Mean ± SEM. *p < 0.05, **p < 0.01, ***p < 0.001, ****p < 0.0001 vs. EBI 0 μg/mL group (one-way ANOVA). **(G)** Representative flow cytometric analysis of annexin V and PI staining of SW620 cells.

To determine the efficacy of EBI as an antitumor agent, we conducted a series of experiments using human CRC cell lines, including SW620, Caco2, and HT29. First, we performed a CCK-8 assay to evaluate EBI’s inhibition of cell proliferation. Cells were treated with various concentrations of EBI (6.25, 12.5, 25, 100, 160, 200, 300, and 400 μg/mL) for 24, 48, or 72 h. Our results show that EBI significantly inhibited the growth of all three CRC cell lines in a concentration- and time-dependent manner (Figures 1A–C). The IC50 values of SW620, HT29, and Caco2 at 24 h were 20.39 ± 0.11 μg/mL, 63.88 ± 1.28 μg/mL, and 78.14 ± 0.04 μg/mL, respectively (Figure 1D). These findings indicate that SW620 cells were the most sensitive to EBI and were used as the pertinent cell model for subsequent studies. We also used flow cytometry analysis to assess the effects of EBI on inducing cell death in SW620 cells. As shown in Figures 1E, G, the population of viable cells significantly decreased in the presence of EBI in a dose-dependent manner. Interestingly, we observed a statistically significant enrichment of double-positive SW620 cells for annexin V and PI, indicating that SW620 cells had undergone apoptotic or necrotic death (Figures 1F, G).

**FIGURE 2:**
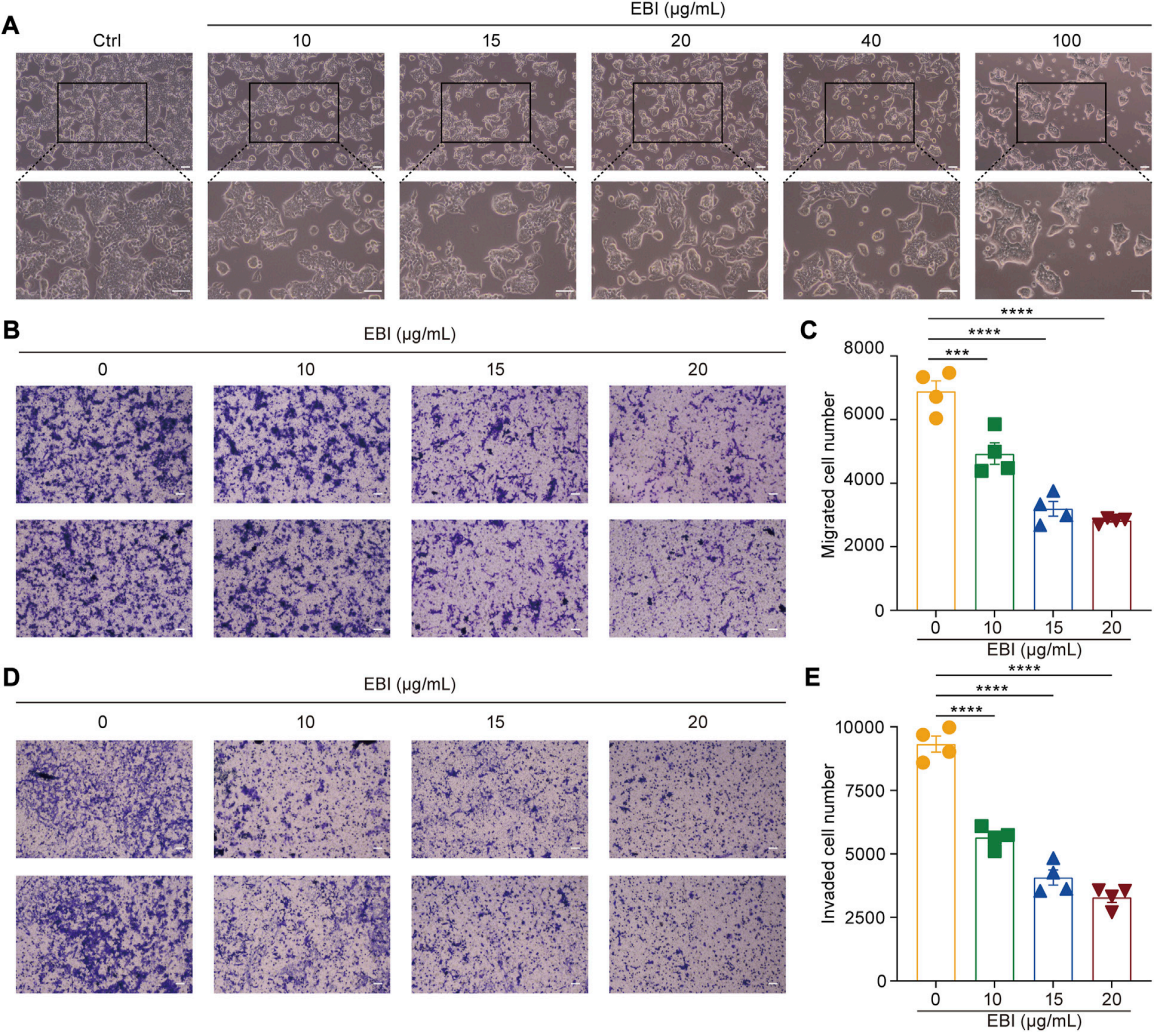
EBI suppresses growth, migration, and invasion of SW620 cells. **(A)** SW620 cells were observed for morphologic changes at 12 h after EBI (10, 15, 20, 40, and 100 μg/mL) treatment. Scale bars indicate 50 μm. **(B,C)** The representative image **(B)** and quantitative analysis **(C)** of migrated cells after exposure to EBI (10, 15, 20, and 40 μg/mL, 24 h). Scale bars indicate 50 μm. **(D,E)** The representative image **(D)** and quantitative analysis **(E)** of invaded cells after exposure to EBI (10, 15, 20, and 40 μg/mL, 24 h). Scale bars indicate 50 μm. Mean ± SEM. ***p < 0.001, ****p < 0.0001 vs. EBI 0 μg/mL group (one-way ANOVA).

Moreover, the photomicrographs of SW620 cells treated with different concentrations of EBI (10, 15, 20, 40, and 100 μg/mL) for 12 h showed that SW620 cells became exceptionally susceptible to cell death with increasing concentration, further supporting the notion that EBI triggers cell death (Figure 2A). Taken together, these results suggest that EBI effectively inhibits the growth and promotes the death of CRC cells *in vitro*.

### 3.2 EBI inhibits CRC cells migration and invasion

Scutellarin, the main active ingredient in EBI, has been demonstrated to have the ability to suppress the migration and invasion of various types of cancer cells (Deng et al., 2018; Lv et al., 2019). Therefore, we sought to determine whether EBI could also inhibit the migration and invasion of SW620 cells. To accomplish this, we conducted a transwell assay to assess the migration and invasion of SW620 cells after 24 h of EBI treatment. Our results indicated a significant reduction in the number of migrating cells in the EBI-treated groups compared to the untreated controls (Figures 2B, C). Similarly, the number of invaded cells in transwells coated with Matrigel was drastically decreased in the EBI-treated cells (Figures 2D, E). These results suggest that EBI possesses the capability to restrain the migration and invasion of SW620 cells.

### 3.3 EBI suppresses tumor growth and metastasis in SW620 xenograft mice

**FIGURE 3:**
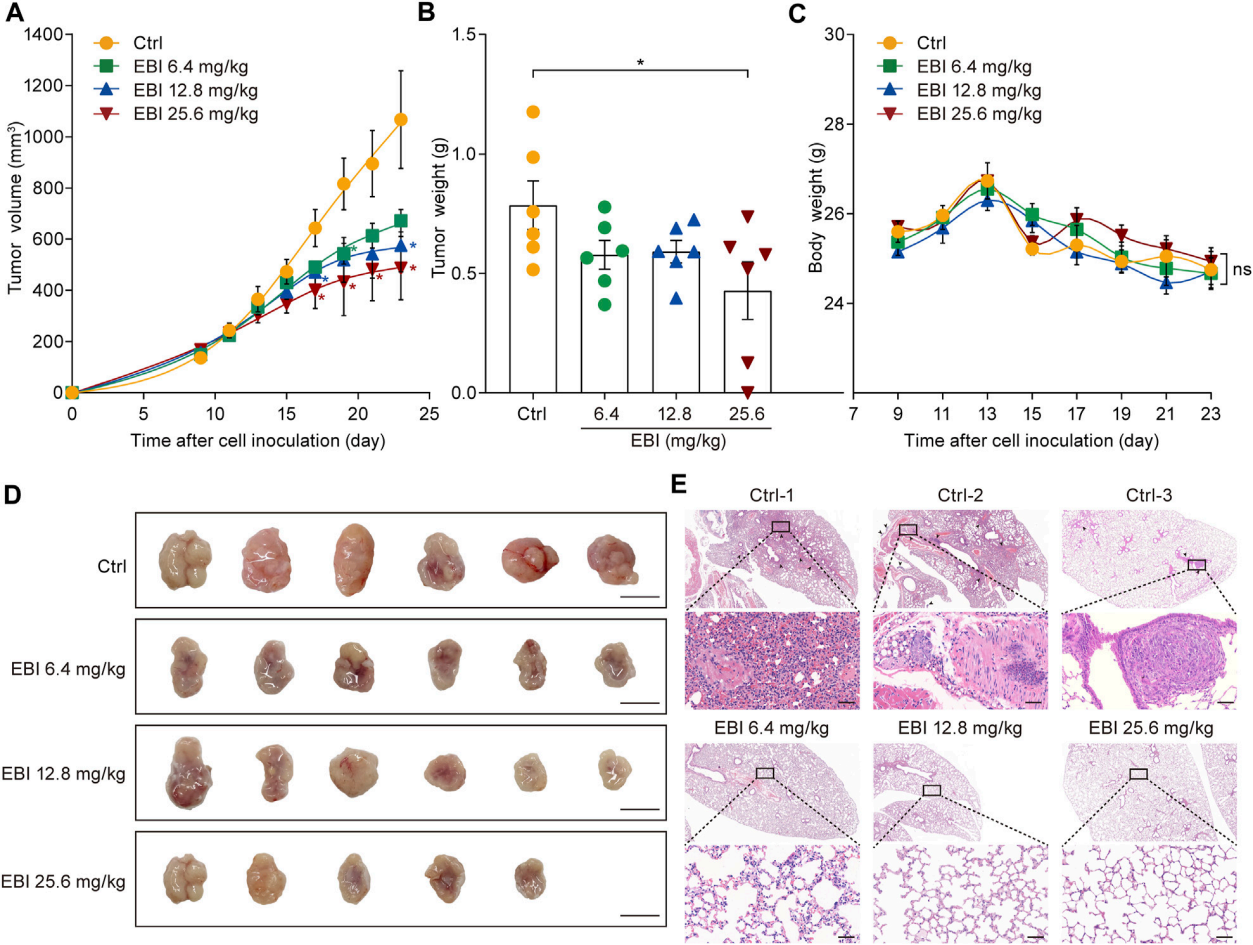
EBI retards tumor growth and lung metastasis in the xenograft SW620 colorectal tumor-bearing mice. **(A,B)** BALB/c-nu mice (*n* = 6) with subcutaneous SW620 cells were i.p. treated with saline or EBI (6.4, 12.8, and 25.6 mg/kg), then the tumor growth was monitored **(A)**, and the weight of tumors was measured after treatment **(B)**. Mean ± SEM. *p < 0.05 vs. control group (*t*-test). **(C)** The body weight curves of SW620-tumor-bearing nude mice during various treatments. Mean ± SEM. **(D)** The morphology of tumor in SW620-tumor-bearing nude mice after various treatments, scale bars indicate 1 cm. **(E)** H&E staining of lung metastasis (indicated by blank arrows) in SW620-tumor-bearing nude mice after treatments with saline or EBI, scale bars indicate 50 μm.

Based on the promising results of our *in vitro* studies, we next investigated the potential therapeutic effect of EBI on CRC progression and metastasis using a xenograft CRC mouse model. SW620 cells were inoculated into the right flank of male nude mice and allowed to grow to approximately 100 mm^3^. Then, EBI was administrated at the ChP 2020 recommended daily human equivalent dosages — 6.4, 12.8, and 25.6 mg/kg once a day for 3 weeks. Compared to the control group, the groups treated with EBI exhibited a significant reduction in tumor volume during the treatment period. Notably, the group treated with EBI at 25.6 mg/kg displayed significant suppression of tumor growth from Day 17 (Figures 3A, D). Furthermore, tumor weight measurements confirmed the therapeutic potential of EBI (Figure 3B). Throughout the EBI treatment, there were no significant differences in body weight among all groups of tumor-bearing mice (Figure 3C), indicating that EBI was well tolerated under the current regimen. Additionally, histological examination of the lungs further demonstrated the therapeutic efficacy of EBI on CRC metastasis, as the groups that received EBI treatment showed a more pronounced reduction in metastatic lung nodules compared to the control group (Figure 3E). Taken together, these results suggest that EBI effectively retards CRC growth and metastasis, highlighting its therapeutic potential.

### 3.4 EBI regulates the expression of necroptosis-related genes in SW620 xenograft mice

**FIGURE 4:**
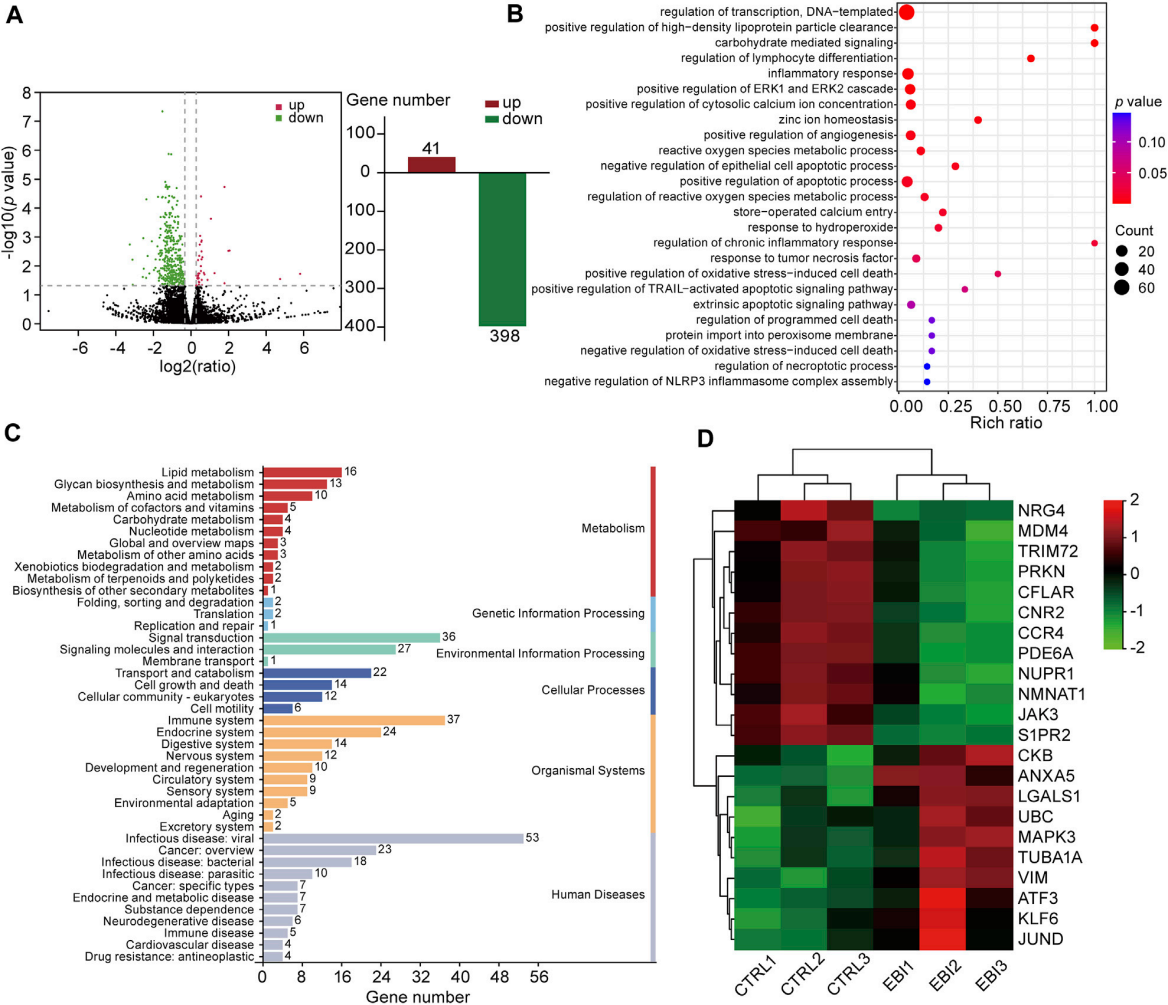
EBI regulates the expression of necroptosis-related genes in SW620 xenograft mice. **(A)** Differentially expressed genes are depicted by a volcano plot. **(B)** The differential genes are functionally clustered based on GO functional enrichment analysis of biological processes among the differentially expressed genes. **(C)** The differential genes were classified into KEGG pathways and grouped into six major categories: metabolism, genetic information processing, environment information processing, cellular process, organismal systems, and human diseases. **(D)** A heat map presenting the gene expression levels of necroptosis-related genes, with red indicating relatively high expression and green indicating relatively low expression.

To understand the molecular mechanism underlying the antitumor activity of EBI in CRC, we investigated whether necroptosis plays a pivotal role in the therapeutic effect of EBI. To achieve this goal, we performed RNA sequencing (RNA-seq) analysis on SW620 xenograft tumors treated with EBI at a dosage of 25.6 mg/kg. As presented in Figure 4A, we identified 439 differentially expressed genes (DEGs), including 41 upregulated and 398 downregulated genes. 439 DEGs were mapped in terms of the gene ontology (GO) database and compared with the whole reference database to access their annotation. The top enriched biological processes were related to programmed cell death, particularly necroptosis (Figure 4B). Moreover, Kyoto Encyclopedia of Genes and Genomes (KEGG) pathway enrichment analysis of our dataset identified the five most significantly enriched pathways, including herpes simplex virus 1 infection, oxytocin signaling pathway, tryptophan metabolism, synthesis and degradation of ketone bodies and estrogen signaling pathway. The enriched pathways were further classified into six major groups: metabolism, genetic information processing, environment information processing, cellular process, organismal systems, and human diseases (Figure 4C). Of these, the sub-group cell growth and death had 14 annotated genes. We also identified necroptosis-related genes based on published studies and the KEGG database (https://www.kegg.jp/kegg/). Notably, EBI treatment upregulated the intra-tumoral transcription of pro-necroptosis genes such as KLF6, LGALS1, and ANXA5, while downregulating the transcription of anti-necroptosis genes such as CFLAR, S1PR2, and TRIM72 (Figure 4D). To visualize the correlation between the identified genes and biological processes, we constructed a Sankey diagram (Supplementary Figure S1). These results indicate that EBI facilitates necroptosis in tumor cells by regulating the expression of necroptosis-related genes.

### 3.5 EBI induces RIPK3/MLKL-mediated necroptosis of CRC cell *in vitro*

**FIGURE 5:**
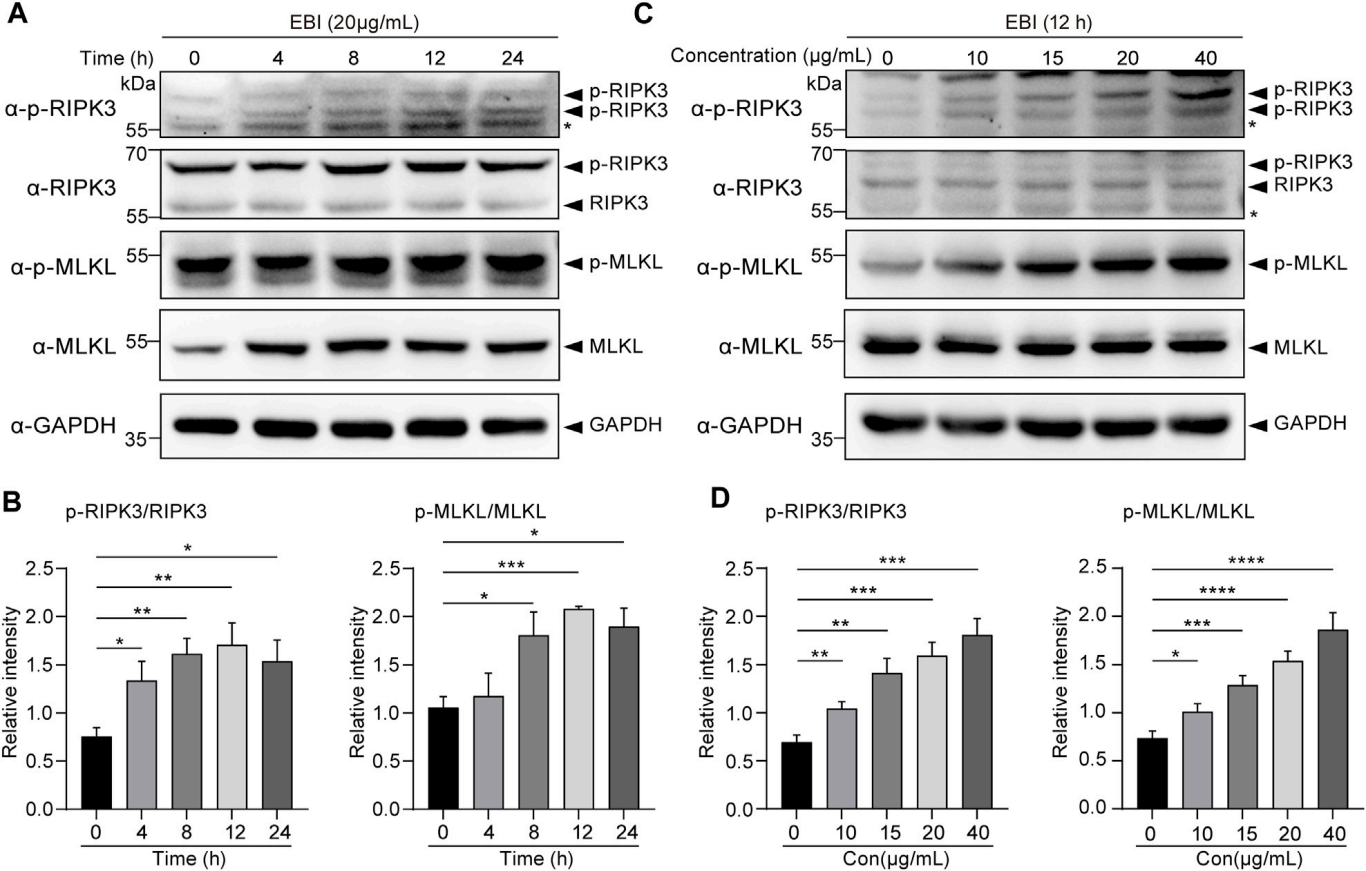
EBI-induced RIPK3/MLKL-mediated necroptosis in SW620 cells *in vitro*. **(A,B)** Immunoblot analysis of p-RIPK3, RIKP3, p-MLKL, and MLKL in SW620 cells treated with EBI (20 μg/mL) for different time intervals (4, 8, 12, and 24 h) **(A)**, and the quantitative analysis of the expression levels of p-RIPK3, RIKP3, p-MLKL, and MLKL, which were normalized to corresponding GAPDH expression levels **(B)**. Mean ± SEM. *p < 0.05, **p < 0.01, ***p < 0.001 vs. 0 h group (*t*-test). **(C,D)** Immunoblot analysis of p-RIPK3, RIKP3, p-MLKL, and MLKL in SW620 cells treated with different concentrations of EBI (10, 15, 20, and 40 μg/mL) for 12 h **(C)**, and the quantitative analysis of the expression levels of p-RIPK3, RIKP3, p-MLKL, and MLKL, which were normalized to corresponding GAPDH expression levels **(D)**. Mean ± SEM. *p < 0.05, **p < 0.01, ***p < 0.001, ****p < 0.0001 vs. EBI 0 μg/mL group (*t*-test).

To confirm our RNA-seq analysis findings, we investigated the activation of necroptosis in SW620 cells during EBI treatment. The cells were treated with 20 μg/mL EBI for different time periods (2, 4, 8, 12, and 24 h), and the expression of the classical necroptotic pathway, namely, RIPK3/MLKL pathway, was assessed. Remarkably, we observed a time-dependent increase in phosphorylation levels of RIPK3 and MLKL following EBI treatment, peaking at 12 h (Figures 5A, B). In addition, when SW620 cells were treated with a series of EBI concentrations (10, 15, 20, and 40 μg/mL) for 12 h, the expression levels of p-RIPK3 and p-MLKL gradually increased with the rising concentration of EBI (Figures 5C, D). These findings suggest that even a low concentration of EBI significantly triggers RIPK3/MLKL-mediated necroptosis in CRC cells *in vitro*, which is consistent with the results obtained from RNA-seq analysis.

### 3.6 EBI exerts its antitumor effects by promoting CRC cell necroptosis

**FIGURE 6:**
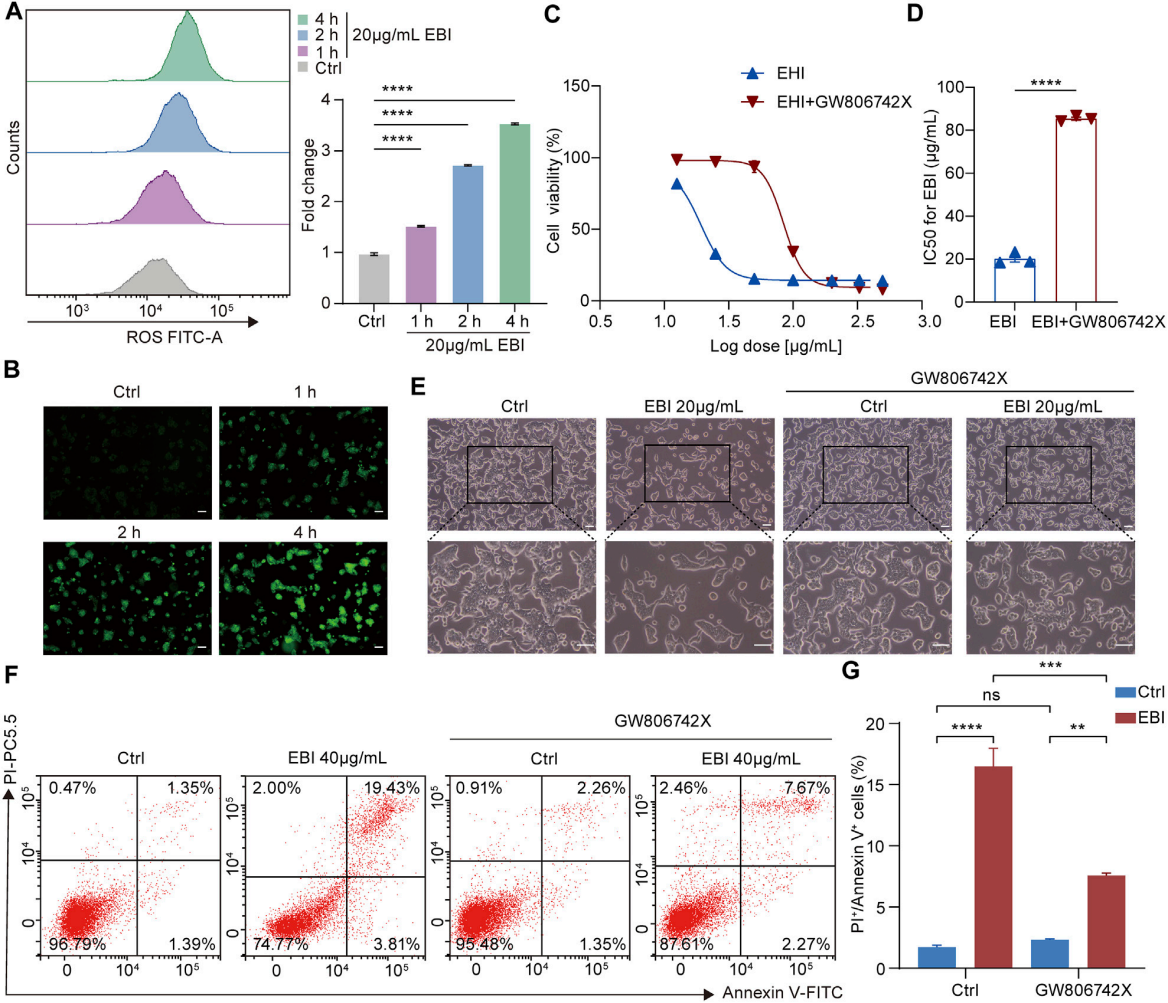
EBI induces ROS generation and exerts antitumor effects by promoting SW620 cell necroptosis *in vitro*. **(A,B)** Time response analysis of EBI treatment on intracellular ROS generation. SW620 cells were treated with EBI (20 μg/mL) for the indicated time periods (1, 2, and 4 h), then incubated with DCFH-DA. The quantitative analysis by flow cytometry **(A)** and the representative fluorescence images obtained via fluorescence microscope are presented in **(B)**. Scale bars indicate 50 μm. Mean ± SEM. ****p < 0.0001 vs. control group (one-way ANOVA). **(C)** The effect of EBI on the viability of SW620 cells for 24 h in the presence or absence of GW806742X, the MLKL inhibitor. **(D)** A comparison of IC50 values (μg/mL) of EBI against SW620 cells for 24 h in the presence or absence of GW806742X. Mean ± SEM. ****p < 0.0001 vs. control group (*t*-test). **(E)** The morphologic changes of SW620 cells at 12 h after EBI treatment with or without GW806742X pretreatment. Scale bars indicate 50 μm. **(F,G)** Flow cytometry analysis of annexin V and PI staining of SW620 cells in the presence or absence of GW806742X. **(F)** Representative flow cytometric analysis of annexin V and PI staining of SW620 cells. **(G)** The quantitative analysis of double-positive cells for annexin V and PI. Mean ± SEM. **p < 0.01, ***p < 0.001, ****p < 0.0001 vs. corresponding group as indicated in the figure (one-way ANOVA).

To investigate the contribution of necroptosis activation to the antitumor efficacy of EBI, we examined the role of reactive oxygen species (ROS) in necrosome formation, as necrosomes are known to disturb ROS homeostasis to impair the energy metabolism of mitochondria, specifically, necrosomal RIPK3 is required for ROS generation (Schulzeosthoff et al., 1992). Moreover, ROS activates RIPK1 autophosphorylation, which enables RIPK1 to recruit RIPK3 and form necrosomes, to form a positive feedback loop driving potent induction of necroptosis (Zhang et al., 2017). We assessed ROS levels in SW620 cells treated with EBI using DCFH-DA assays and found that EBI augmented ROS levels in a time-dependent manner (Figures 6A, B), indicating that EBI activates necroptosis in SW620 cells. We further assessed the antitumor effects of EBI in the presence or absence of the MLKL inhibitor GW806742X (Hildebrand et al., 2014). We observed that the inhibition of SW620 growth by EBI was limited by pretreatment with GW806742X, as shown in Figures 6C, D. Specifically, the IC50 of SW620 increased from 20.15 ± 1.56 μg/mL to 85.41 ± 0.74 μg/mL. A similar trend was observed in Figure 6E, the photomicrographs of SW620 cells treated with EBI for 12 h in the presence or absence of GW806742X showed that the cytotoxicity of EBI against SW620 cells was attenuated after the pretreatment with the inhibitor. Flow cytometry analysis revealed that the population of annexin V and PI double-positive SW620 cells was prominently reduced in response to co-treatment of EBI and GW806742X (Figures 6F, G), suggesting that EBI promotes SW620 cell necroptosis, whereas pharmacological MLKL blockade reverses this effect. To further confirm the role of necroptosis induction in the anti-CRC effects of EBI, MLKL expression was silenced with small interfering RNA (siRNA). RT-qPCR analysis revealed a significant decrease in the relative MLKL mRNA levels of SW620 cells following transfection with MLKL-siRNA (Supplementary Figure S2C). As demonstrated in Supplementary Figure S2A, the knockdown of MLKL reversed the inhibitory effects of EBI on SW620 cell proliferation. Furthermore, MLKL knockdown notably elevated the IC50 value to 106.4 ± 1.22 μg/mL. Taken together, these results emphasize the vital role of necroptosis activation in the CRC inhibitory activity of EBI.

**FIGURE 7:**
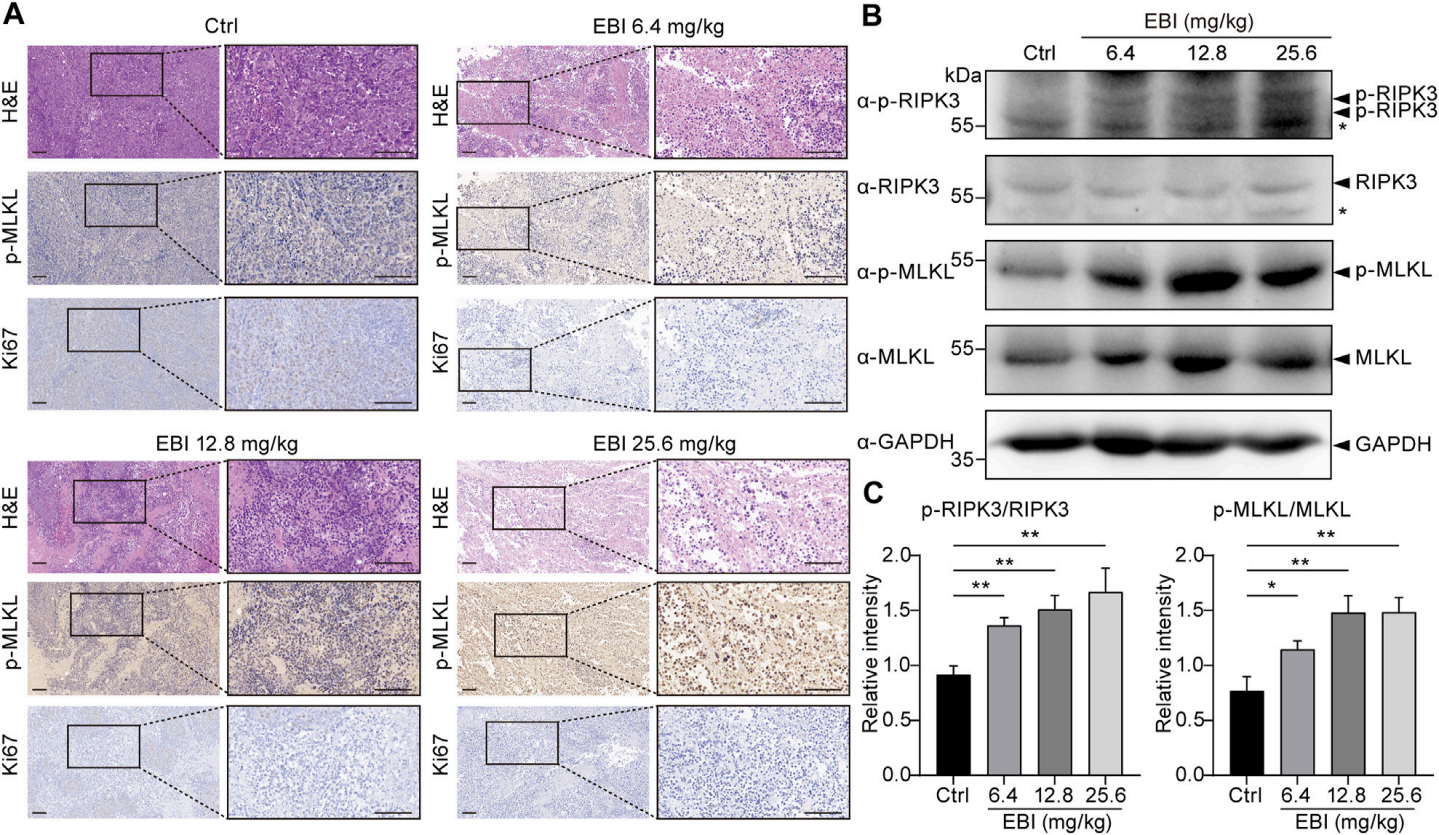
EBI triggers RIPK3/MLKL-mediated necroptosis in xenograft SW620 tumor-bearing mice *in vivo*. BALB/c-nu mice at 1-week post-implantation with SW620 cells were treated with saline or EBI (6.4, 12.8, and 25.6 mg/kg, i.p.) for 3 weeks. **(A)** Representative images of H&E and immunohistochemical stained sections with phospho-MLKL (p-MLKL) antibody or Ki67 antibody. Scale bars indicate 50 μm. **(B,C)** Immunoblot analysis of p-RIPK3, RIKP3, p-MLKL, and MLKL in tumors from xenograft SW620 tumor-bearing mice after the treatment **(B)**, and the quantitative analysis of the expression levels of p-RIPK3, RIKP3, p-MLKL, and MLKL, which were normalized to corresponding GAPDH expression levels **(C)**. Mean ± SEM. *p < 0.05, **p < 0.01, vs. control group (*t*-test).

Finally, we attempted to verify if necroptosis was triggered by EBI in xenograft SW620 tumor-bearing mice *in vivo*. The H&E-stained sections of EBI-treated groups displayed markedly enhanced tumor necrosis compared to the control group (Figure 7A), and the phosphorylation level of MLKL in necrotic areas of EBI-treated tumors elevated substantially in a dose-dependent manner, while only modest amount of MLKL phosphorylation was detected in the control group (Figure 7A). These results suggest that the necrotic areas identified in H&E-stained sections could be attributed to necroptosis. Moreover, the level of Ki67, a widely used proliferation marker, was significantly reduced (Figure 7A). Western blot analysis also showed that the protein expression levels of p-RIPK3 and p-MLKL in the tumor site were significantly upregulated after treatment with different doses of EBI in mice (Figures 7B, C). Thus, these findings displayed the heightened antitumor response of EBI, with hindered CRC proliferation, which function through necroptosis induction. Collectively, these findings support the notion that EBI potentiates necroptosis of CRC to exert antitumor efficiency in *in vitro* and *in vivo* studies.

**FIGURE 8:**
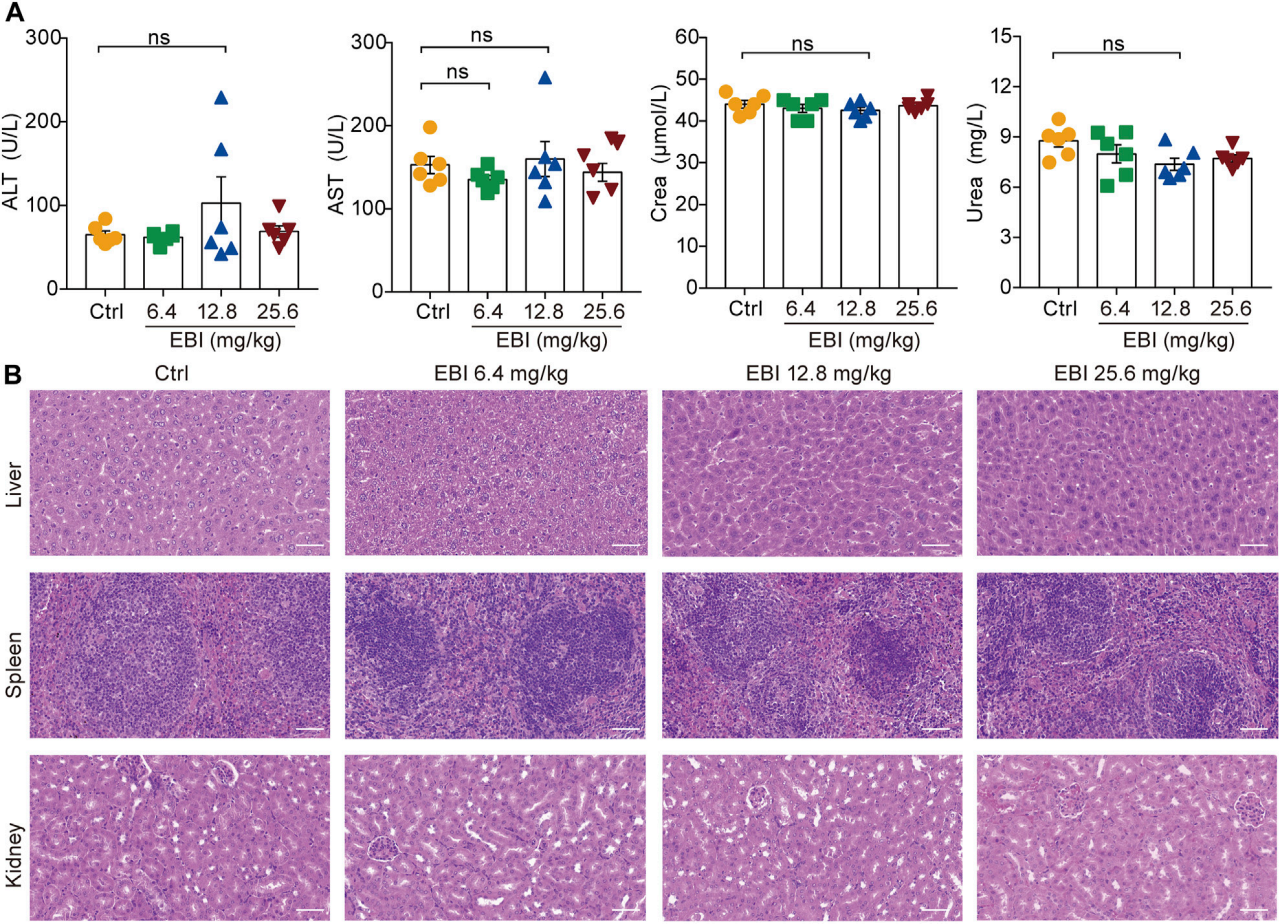
EBI exhibits low-toxicity in xenograft SW620 tumor-bearing mice. **(A)** The levels of the serum biochemical parameters ALT, AST, Crea, and Urea. Mean ± SEM (one-way ANOVA). **(B)** Representative images of pathological examination of the major organs (the liver, spleen, and kidney). The tissue sections were stained with H&E. Scale bars indicate 50 μm.

It is noteworthy that in the current treatment regimen, all EBI dosages did not result in increased levels of serum biochemical parameters (ALT, AST, Crea, and Urea), indicating that EBI administration does not cause severe toxicity or side effects in mice (Figure 8). These findings confirm the safety of EBI and underscore the importance of EBI-induced necroptosis in mediating its antitumor effects in CRC, at least partially via the RIPK3/MLKL-mediated pathway.

## 4 Discussion

**FIGURE 9:**
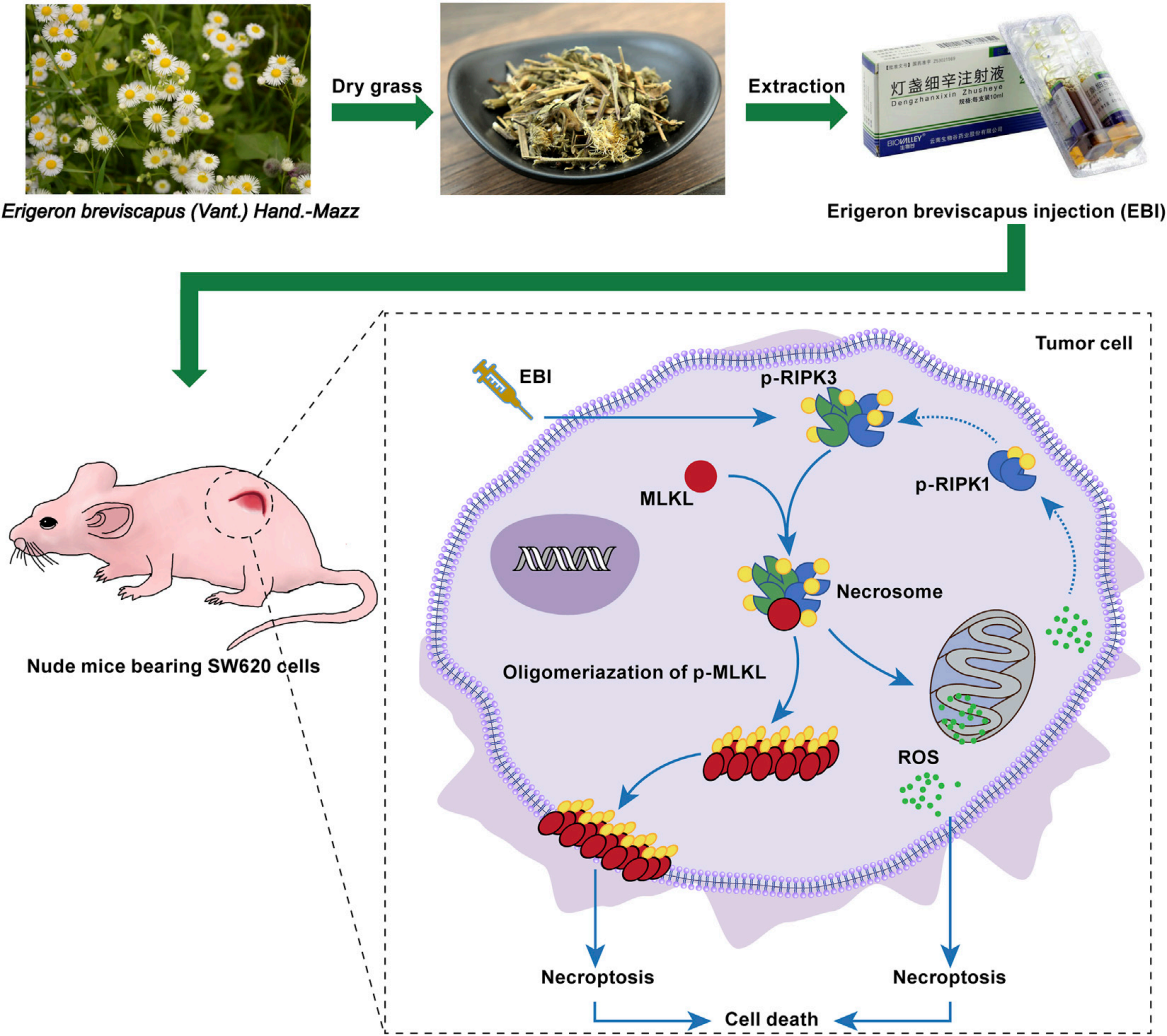
EBI exhibits potent antitumor effects in xenograft SW620 tumor-bearing mice via inducing RIPK3/MLKL-mediated necroptosis.

EBI, a widely used clinical therapy for cardiovascular and cerebrovascular diseases, has demonstrated excellent safety with minimal adverse reactions. Recent studies have also identified its potential as an anticancer agent, with active compounds such as SCU exhibiting potent anticancer activity against various types of cancer, including colorectal cancer (Zhu et al., 2017), bladder cancer (Lv et al., 2019), hepatocellular carcinoma (Pang et al., 2022), breast cancer (Hou et al., 2017), cervical cancer (You et al., 2017), prostate cancer (Gao et al., 2017), NSCLC (Cao et al., 2019) and others. Moreover, phenolic compounds, another major constituent in EBI, have been shown to exert anticancer effects (Pang et al., 2022). In this study, we investigated for the first time that EBI function as a safe necroptosis inducer for anti-CRC therapy (Figure 9). *In vitro* experiments showed that EBI effectively impeded the growth of CRC cells and induced cell death. Furthermore, EBI treatment at low concentrations (10 μg/mL) suppressed the migration and invasion of SW620 cells. Notably, EBI exhibited potent anticancer and anti-metastatic effects in mice bearing SW620 cancer cells at human equivalent dosages recommended by the ChP 2020.

While chemotherapy, surgery, and radiation therapy are the primary pillars of cancer treatment, targeted therapy and immunotherapy are emerging as promising fields. However, the emergence of drug resistance and intolerable side effects from current treatments underscores the critical need for new, safe, and effective drugs.

Apoptosis evasion is a hallmark of cancer and can promote tumor growth, progression, and drug resistance (Weiss et al., 2022). Therefore, the discovery of new natural compounds that induce non-apoptotic programmed cell death may have significant therapeutic implications. Necroptosis, a form of programmed cell death, is closely associated with pathological conditions (Khan et al., 2021). Recent evidence has highlighted the potential of targeting necroptosis, especially for tumors resistant to apoptosis. For example, studies have shown that using a second mitochondria-derived activator of caspase (SMAC) mimetic can induce necroptosis and overcome drug resistance in caspase-8-deficient CRC mouse models (He et al., 2017). Additionally, recent studies have revealed that the RNA editing enzyme ADAR1 is a determinant of resistance to immune checkpoint blockade (ICB) therapy. Depletion or mutation of ADAR1 can activate Z-DNA-binding protein 1 (ZBP1), ultimately leading to RIPK3-mediated necroptosis. Zhang et al. (2022) used the small molecule CBL0137 to activate ZBP1 and reverse ICB unresponsiveness in melanoma mice.

Recently, there has been growing interest in identifying natural products that can induce tumor necroptosis without causing toxicity. For example, shikonin (SHK), a natural naphthoquinone derivative isolated from *Lithospermum erythrorhizon*, has been reported to induce necroptosis in MCF-7 breast cancer cells with high expression of the anti-apoptotic protein B cell lymphoma 2 (Su et al., 2016). Other studies have found that SHK could trigger necroptosis in glioblastoma and osteosarcoma cells (Fu et al., 2013; Huang et al., 2013). These findings suggest that developing natural products capable of inducing necroptosis may represent a novel strategy to overcome tumor resistance.

The present study provides evidence that EBI induces necroptosis in colorectal cancer cells. To investigate the underlying mechanism, RNA-seq was performed on tumors from EBI-treated mice. Our results revealed that EBI activates a complex signaling network leading to necroptosis in cancer cells. Notably, EBI treatment was associated with a reduction in the expression of genes that promote poor necroptosis, such as CFLAR, which encodes for the caspase 8 antagonist protein c-FLIP. Cells with downregulated CFLAR expression were found to be sensitive to necroptosis, highlighting its importance in the process (Wang et al., 2017). Conversely, the expression of certain genes that promote necroptosis, including KLF6, LGALS1, ANXA5, and others, was significantly upregulated after EBI treatment. Galectin-1 (LGALS1) release is a common feature of inflammatory cell death, including necroptosis (Russo et al., 2021), while KLF6 downregulation has been shown to rescue the death of bortezomib-resistant multiple myeloma cells (Raninga et al., 2016). Previous studies have shown that SCU can overcome resistance to cisplatin-induced apoptosis and autophagy in NSCLC by activating the ERK/p53 and c-met/Akt signaling pathways (Sun et al., 2018). Therefore, further research could explore whether EBI has the potential to reverse tumor resistance by inducing cell necroptosis. It is noteworthy that SCU, as a potential chemosensitizer, has shown promising therapeutic effects, particularly in platinum-resistant tumor cells (Sun et al., 2018; Sun et al., 2021). Building upon the findings of the present study, these results suggest that further investigation of the combination of EBI with other apoptosis-inducing agents, such as chemotherapeutic drugs, could be a prospective strategy to enhance chemotherapy efficacy and overcome drug resistance.

Protein phosphorylation is a crucial mechanism that regulates various cellular processes. It is well established that RIP1, RIP3, and MLKL are key components in the necroptosis pathway and undergo phosphorylation during the process. The phosphorylation status of RIPK3 and MLKL is widely used as a marker to assess necroptosis (Weinlich et al., 2017). Our study indicates that the protein expression of p-RIPK3 and p-MLKL was significantly upregulated in SW620 cells following EBI treatment, reaching its peak at 12 h post-treatment. Moreover, the expression levels of p-RIPK3 and p-MLKL were positively correlated with the concentration of EBI. Additionally, our histopathological analysis on tumors of mice treated with different doses of EBI demonstrated that treatment with EBI led to a dose-dependent increase in p-MLKL expression and a decrease in Ki67 expression. These findings suggest that EBI can effectively induce RIPK3/MLKL phosphorylation *in vitro and in vivo*. However, cell necroptosis is a complex process involving multiple genes/proteins. Recent studies have reported that ZBP-1 regulates necroptosis in a manner independent of RIPK1-mediated necroptosis, suggesting a new mechanism of action (Baik et al., 2021; Jiao et al., 2022). Therefore, the involvement of other pathways in necroptosis cannot be ruled out. Furthermore, EBI is composed of multiple active compounds, and there has been evidence demonstrating the inhibitory effects of SCU on tumor migration and invasion (Ke et al., 2017; Lv et al., 2019). Therefore, the net effect of EBI seen in this study seems based on a drug complex system (Kong et al., 2020), in which the multiple compounds and its metabolites interact with molecular targets and other biological components to cause synergistic effect. At least in part, this effect is mediated through the RIPK3/MLKL-mediated pathway.

Interestingly, ROS production is RIP3 dependent (Zhang et al., 2017) and can promote necroptosis (Schulzeosthoff et al., 1992), forming a positive feedback circuit. We successfully detected an increase in intracellular ROS production in SW620 cells following EBI treatment using DCFH-DA, confirming the ability of EBI to induce ROS generation. Furthermore, we employed GW806742X, a specific MLKL inhibitor, to validate the contribution of necroptosis in the anti-CRC effects of EBI (Hildebrand et al., 2014). Our results demonstrated that GW806742X increased the IC50 values of EBI against SW620 cells and mitigated the increase in the proportion of double-positive SW620 cells for PI and annexin V induced by EBI. In addition, we confirmed the importance of necroptosis induction in the anti-CRC effects of EBI through MLKL silencing using siRNA. However, to provide more conclusive evidence for the involvement of necroptosis in EBI’s antitumor effects, we recommend using MLKL-deficient mice in future studies. Finally, we evaluated the safety and tolerability of EBI, which showed excellent safety and tolerability consistent with clinical results.

The present study provides compelling evidence for the potent anticancer properties of EBI, which induces necroptosis in colorectal cancer. These findings offer novel therapeutic opportunities for cancer treatment. Given the established safety and clinical utility of EBI, our results further validate its potential as an effective anticancer agent, demonstrating the feasibility of repurposing EBI for cancer therapy.

## Data Availability

The datasets presented in this article are not readily available because of patient confidentiality. Requests to access the datasets should be directed to the corresponding authors.

## Abbreviations

AST, aspartate aminotransferase; ALT, alanine aminotransferase; ChP 2020, 2020 edition of Pharmacopoeia of the People’s Republic of China; Crea, creatinine; CRC, colorectal cancer; DCFH-DA, dichlorodihydrofluorescein diacetate; EBI, Erigeron breviscapus injection; EHM, *Erigeron breviscapus (Vant.) Hand. Mazz*; GO, Gene Ontology; H&E, hematoxylin and eosin; ICB, immune checkpoint blockade; KEGG, Kyoto Encyclopedia of Genes and Genomes; LGALS1, galectin-1; MLKL, mixed lineage kinase domain-like protein; NSCLC, non-small cell lung cancer; PFA, paraformaldehyde; PI, propidium iodide; RIPK1, receptor-interacting protein kinase 1; RIPK3, receptor-interacting protein kinase 3; ROS, reactive oxygen species; SCU, scutellarin; SHK, shikonin; Urea, urea nitrogen; ZBP1, Z-DNA-binding protein 1.
