# Corrigendum: Induction of RIPK3/MLKL-mediated necroptosis by *Erigeron breviscapus* injection exhibits potent antitumor effect

**DOI:** 10.3389/fphar.2023.1271953

**Published:** 2023-08-28

**Authors:** Xiuping Guo, Rui Li, Jinjin Cui, Chujuan Hu, Haoyang Yu, Ling Ren, Yangyang Cheng, Jiandong Jiang, Xiao Ding, Lulu Wang

**Affiliations:** ^1^ Institute of Medicinal Biotechnology, Chinese Academy of Medical Science and Peking Union Medical College, Beijing, China; ^2^ State Key Laboratory of Phytochemistry and Plant Resources in West China, Kunming Institute of Botany, Chinese Academy of Sciences, Kunming, China

**Keywords:** *Erigeron breviscapus* injection, Dengzhanxixin, *Erigeron breviscapus* (Vant.) Hand.-Mazz, colorectal cancer, necroptosis, drug resistance

In the published article, there was an error in Figure 2 as published. The upper panels in Figures 2B, D were inadvertently misused during the final assembly of Figure 2. The corrected Figure 2 appear below.

**FIGURE 2:**
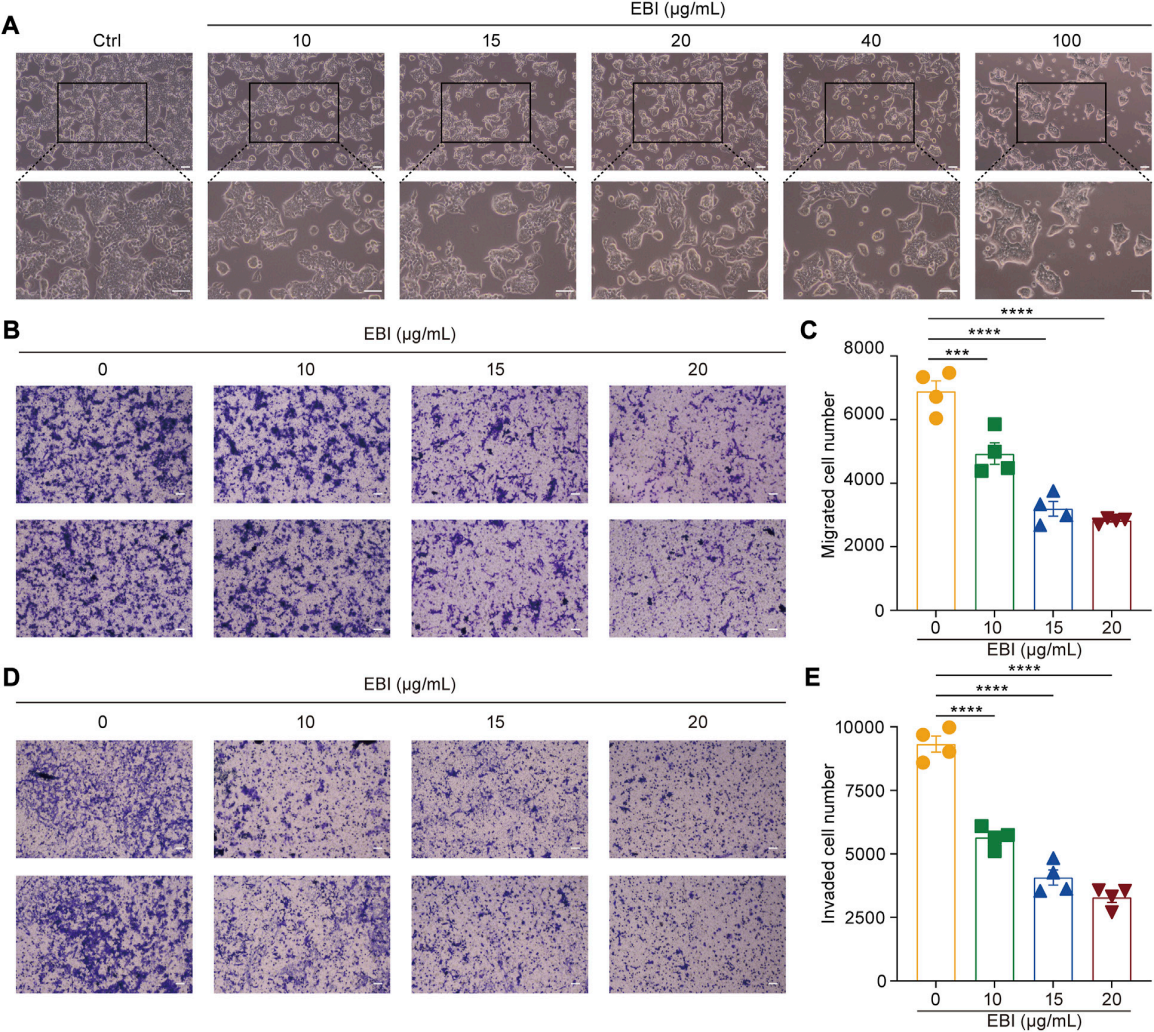
EBI suppresses growth, migration, and invasion of SW620 cells. **(A)** SW620 cells were observed for morphologic changes at 12 h after EBI (10, 15, 20, 40, and 100 μg/mL) treatment. Scale bars indicate 50 μm. **(B, C)** The representative image **(B)** and quantitative analysis **(C)** of migrated cells after exposure to EBI (10, 15, 20, and 40 μg/mL, 24 h). Scale bars indicate 50 μm. **(D, E)** The representative image **(D)** and quantitative analysis **(E)** of invaded cells after exposure to EBI (10, 15, 20, and 40 μg/mL, 24 h). Scale bars indicate 50 μm. Mean ± SEM. ***p < 0.001, ****p < 0.0001 vs. EBI 0 μg/mL group (one-way ANOVA).

The authors apologize for this error and state that this does not change the scientific conclusions of the article in any way. The original article has been updated.

