# Supplementary figures and images for: Induction of RIPK3/MLKL-mediated necroptosis by Erigeron breviscapus injection exhibits potent antitumor effect
Source: Front Pharmacol. 2023 Jun 16;14:1219362. doi: 10.3389/fphar.2023.1219362 (PMC10311648; doi:10.3389/fphar.2023.1219362)

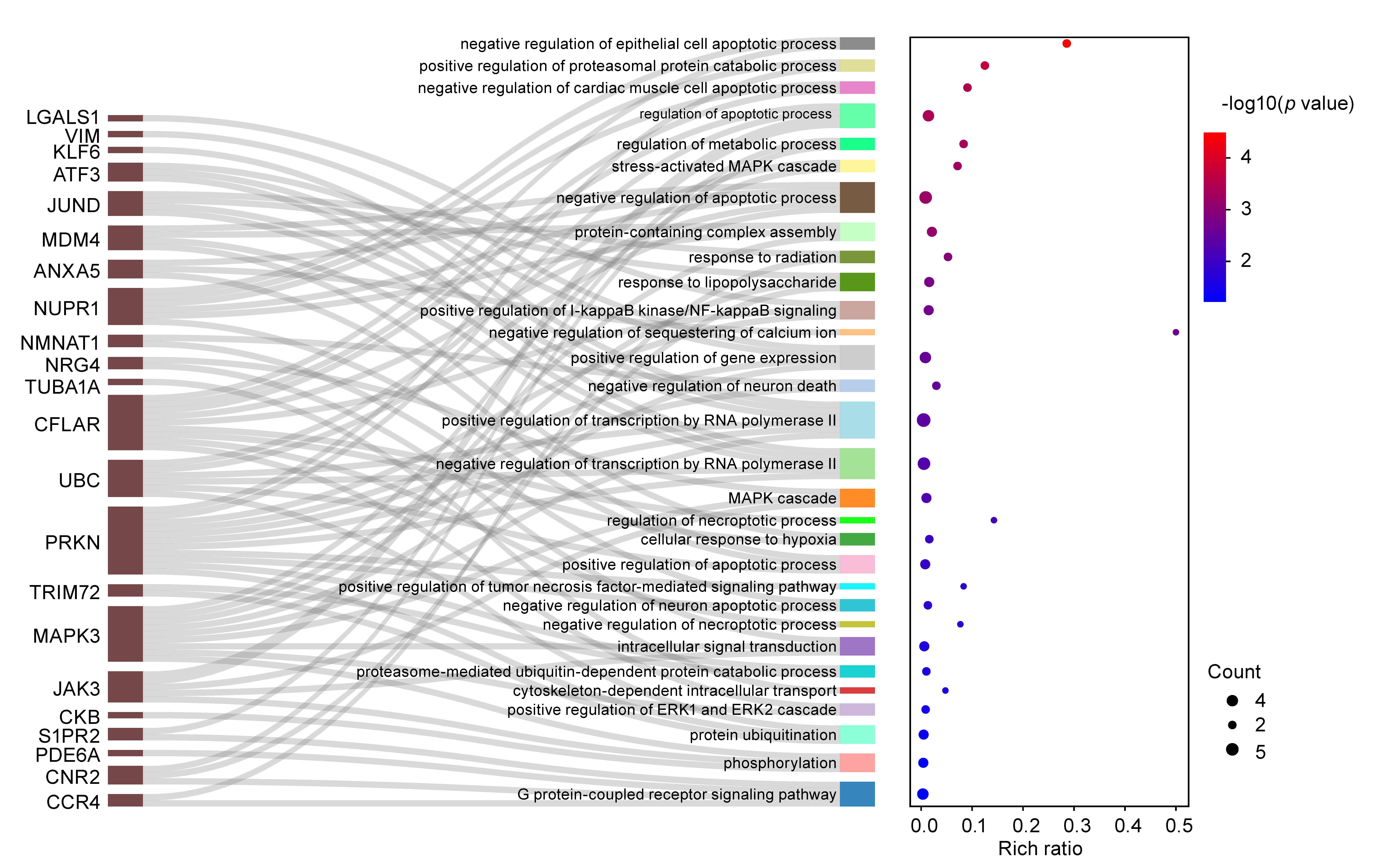

Supplement: Supplementary file 1 [file Image1.JPEG]

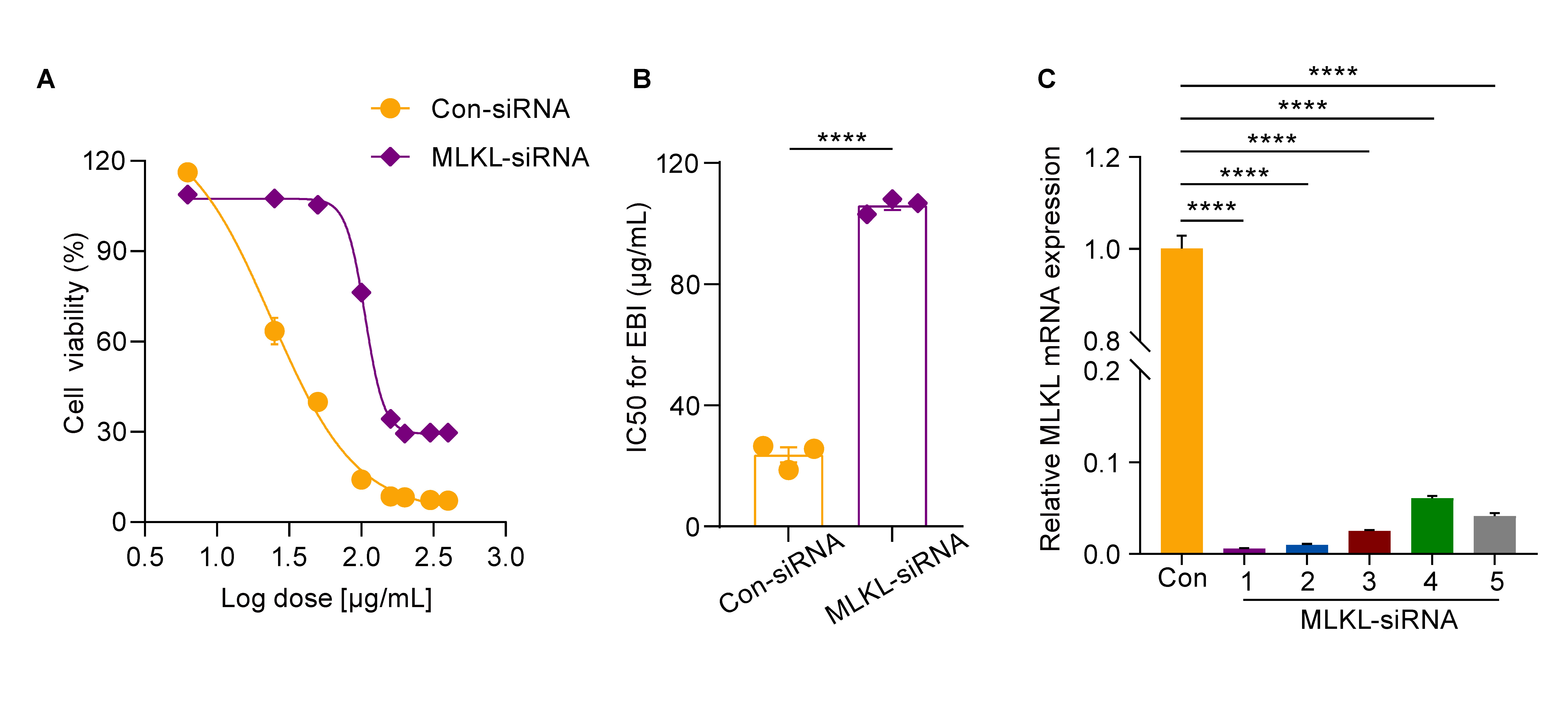

Supplement: Supplementary file 2 [file Image2.JPEG]
